# Supplementary material for: Differential HIV-1 Proviral Defects in Children vs. Adults on Antiretroviral Therapy
Source: bioRxiv. 2025 May 27:2025.05.23.655786. Preprint. [Version 1] doi: 10.1101/2025.05.23.655786 (PMC12154797; doi:10.1101/2025.05.23.655786)

bioRxiv preprint doi: https://doi.org/10.1101/2025.05.23.655786; this version posted May 27, 2025. The copyright holder for this preprint (which was not certified by peer review) is the author/funder. This article is a US Government work. It is not subject to copyright under 17 USC 105 and is also made available for use under a CC0 license.

SUPPLEMENTAL TABLES AND FIGURES

Table S1. PCR primers used for near full-length HIV PCR amplification.

Table S2. PCR conditions for near full-length sequencing of HIV proviruses.

Figure S1. Various proviral structures resulting from HIV-1 near full-length (NFL) amplification (~9kb). A. Reference HIV-1 genome (accessed from https://www.hiv.lanl.gov). B. Intact HIV-1 genome. C. Genome with large internal deletion. C1. Genome with 3' deletion. C2. Genome with 5' deletion. C3. Genome with both 5' and 3' deletion. D1. Small internal deletions. D2. Insertions. D3. Defects in regulatory elements including major splice donor site, packaging signal, *gag* start codon, *rev* response element. D4. Premature stop codons resulting from hypermutation.

Supplemental Table 1. PCR primers used for near full-length HIV PCR amplification

Genome region	Primer location (HXB2 reference)	Sequence (5′-3′)
5′ LTR_U5	623+	AAATCTCTAGCAGTGGCGCCCGAACAG
gag leader	642+	CCGAACAGGGACBHGAAAGCGAA
3′ LTR_U5	9604-	TGAGGGATCTCTAGTTACCAGAGTC
3′ LTR_U5	9662-	GCACTCAAGGCAAGCTTTATTGAGGCTTA

Supplemental Table 2. PCR conditions for near full-length sequencing of HIV proviruses.

Polymerase	PCR1 conditions	PCR2 conditions
Ranger	1. 95°C for 2 min<br>2. 98°C for 10 sec<br>3. 61.5°C for 10 min<br>4. 72°C for 10 min<br>5. Go to Step 2, 30x cycles<br>6. 4°C hold	1. 95°C for 2 min<br>2. 98°C for 10 sec<br>3. 65.5°C for 10 min<br>4. 72°C for 10 min<br>5. Go to Step 2, 30x cycles<br>6. 4°C hold
SuperFi II	1. 98°C for 3 min<br>2. 98°C for 10 sec<br>3. 61.5°C for 30 sec<br>4. 72°C for 5 min<br>5. Go to Step 2, 30x cycles<br>6. 72°C for 5 min<br>7. 4°C hold	1. 98°C for 3 min<br>2. 98°C for 10 sec<br>3. 65.5°C for 30 sec<br>4. 72°C for 5 min<br>5. Go to Step 2, 30x cycles<br>6. 72°C for 5 min<br>7. 4°C hold

bioRxiv preprint doi: https://doi.org/10.1101/2025.05.23.655786; this version posted May 27, 2025. The copyright holder for this preprint (which was not certified by peer review) is the author/funder. This article is a US Government work. It is not subject to copyright under 17 USC 105 and is also made available for use under a CC0 license.

Supplemental Figure 1

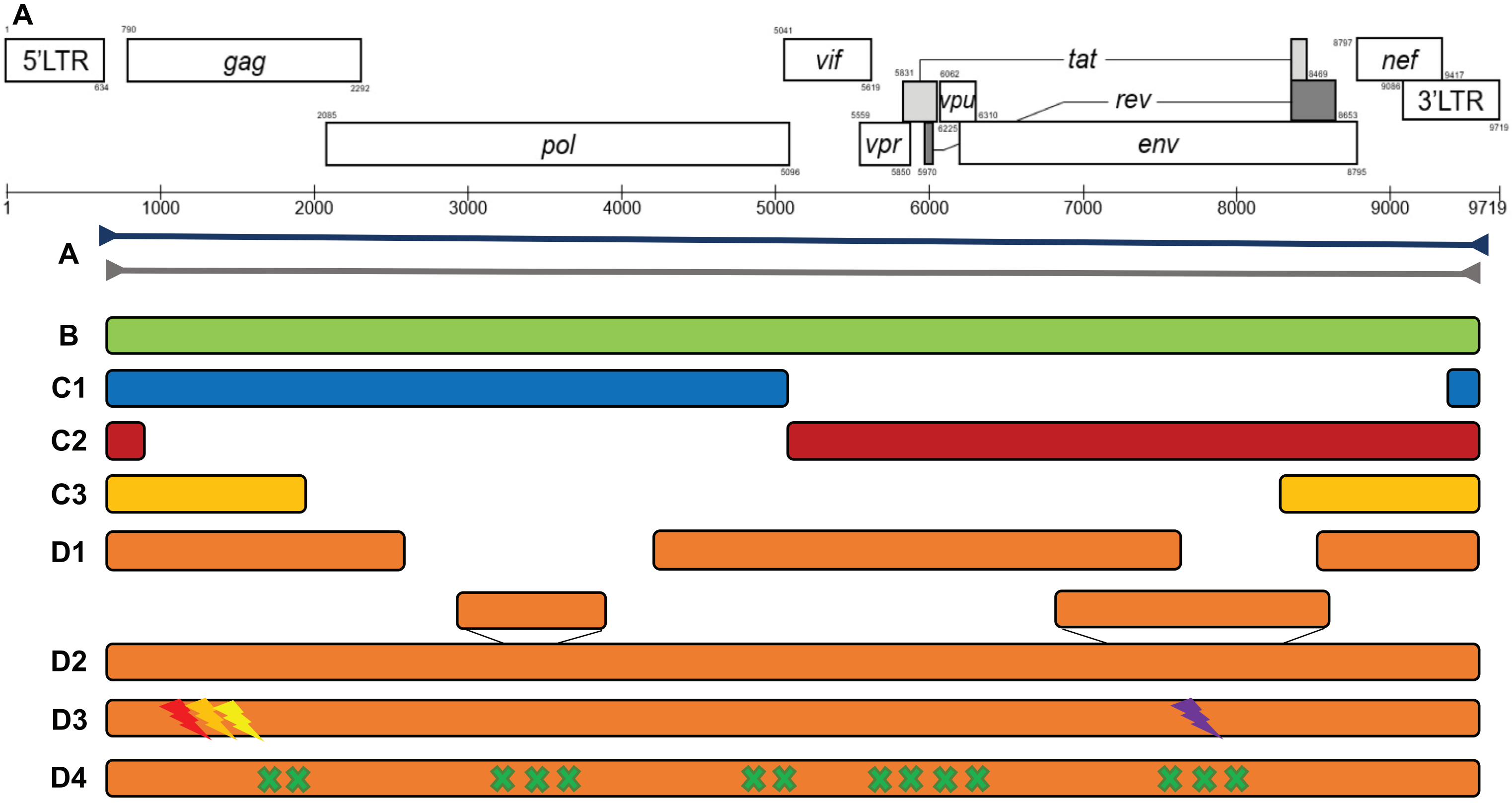

Supplement: Supplement 1 [file NIHPP2025.05.23.655786v1-supplement-1.pdf]
